# Integrated transcriptomics and metabolomics reveal multi-target mechanisms of tannins against *Clostridium perfringens* and necrotic enteritis

**DOI:** 10.1186/s40104-025-01228-3

**Published:** 2025-07-14

**Authors:** Huiping Xu, Lu Gong, Yuming Guo

**Affiliations:** State Key Laboratory of Animal Nutrition and Feeding, College of Animal Science and Technology, China Agricultural University, Beijing, 100193 China

**Keywords:** *Clostridium perfringens*, Intestinal epithelial cells, Metabolome, Necrotic enteritis, Tannins, Transcriptome

## Abstract

**Background:**

*Clostridium perfringens* is a pathogen that secretes multiple toxins, impacting humans and animals. It can cause intestinal diseases such as necrotic enteritis. Although tannins inhibit *C. perfringens* proliferation, the precise underlying mechanisms are unclear.

**Objective:**

This study integrated transcriptomics and metabolomics to systematically investigate the mechanism by which tannins, specifically pentagalloylglucose (PGG) and tannic acid (TA), inhibit *C. perfringens* and potential pathways to alleviate infection in vivo.

**Results:**

Ion concentration measurements, flow cytometric analysis, and transmission electron microscopy revealed that PGG and TA damaged the cell membrane structure of *C. perfringens*, triggering cytoplasmic content leakage. Additionally, PGG and TA significantly affected *C. perfringens* at the transcriptional and metabolic levels. Bioinformatics analysis revealed that PGG and TA induced amino acid restriction, disrupted energy metabolism, and impeded the ability of *C. perfringens* to sense and respond to the external environment. In an in vitro* C. perfringens-*infected intestinal cell model, PGG and TA bound α toxin, significantly reduced the mRNA expression of inflammatory factors, and improved intestinal barrier function and cell viability. Compared to PGG, TA exhibited stronger inhibitory activity against *C. perfringens* and binding to α toxin. In vivo, PGG and TA alleviated *C. perfringens*-induced weight loss in mice, improved intestinal villi morphology, and reduced intestinal inflammation and tight junction gene dysregulation.

**Conclusion:**

These findings indicate that tannins inhibit *C. perfringens*, improve gut tissue integrity and reduce inflammation, demonstrating their multi-target effects of resisting intestinal diseases caused by harmful bacteria. This offers new insights for plant polyphenol-based strategies against necrotic enteritis.

**Graphical Abstract:**

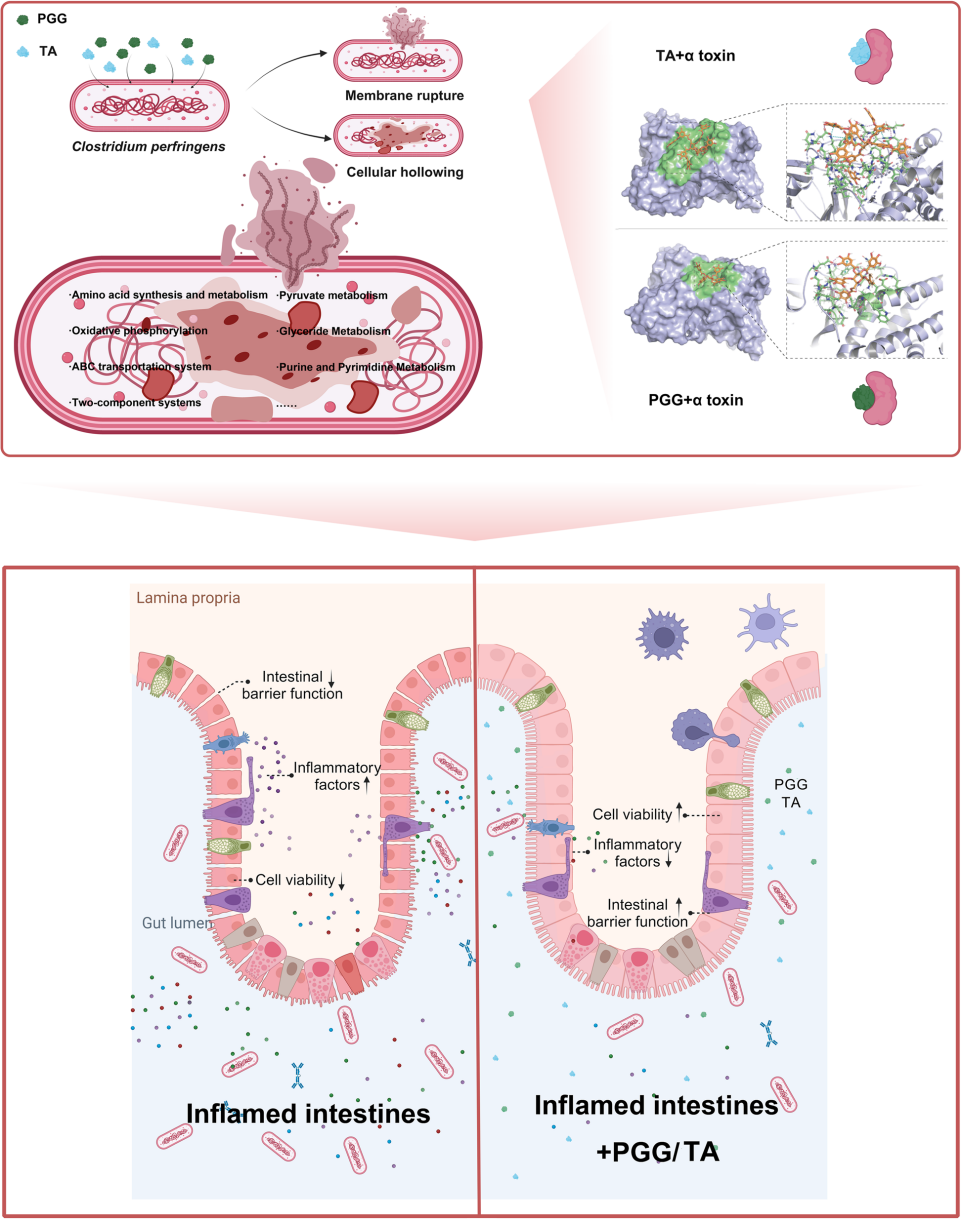

**Supplementary Information:**

The online version contains supplementary material available at 10.1186/s40104-025-01228-3.

## Background

*Clostridium perfringens*, a Gram-positive anaerobic pathogen, is associated with health risks for humans and livestock. In humans, it is a leading cause of foodborne illness, with approximately one million cases annually in the United States [1], primarily linked to contaminated meat and poultry products. In many developed countries, foodborne illness caused by *C. perfringens* ranks second [2]. Its enterotoxins, such as α toxin and NetB toxin, disrupt intestinal barrier integrity, leading to severe gastroenteritis and systemic infections. In poultry, *C. perfringens* infections result in necrotic enteritis, causing substantial economic losses due to high mortality rates and reduced productivity [3]. Meanwhile, the overuse of antibiotics in agriculture has contributed to the emergence of multidrug-resistant *C. perfringens* strains, limiting therapeutic options [4].

Tannins are naturally occurring phenolic compounds found in various plants. Tannins exhibit antibacterial, antioxidant, and anti-inflammatory properties, inhibiting a wide range of harmful bacteria and playing a key role in maintaining intestinal health. In animal husbandry, a tannin-based diet inhibits *C. perfringens* growth in animals and reduces the effects of necrotic enteritis in broilers [5]. Meanwhile, in humans, consuming polyphenols in green tea concentrates for four consecutive weeks significantly reduces the relative abundance of *C. perfringens* in the intestines [6]. Additionally, in contrast to antibiotics such as bacitracin and avilamycin, *C. perfringens* does not develop resistance to tannins [7]. However, while previous studies have demonstrated the inhibitory and toxin-producing activities of different tannins against* C. perfringens* [8–10], specific mechanistic insights are lacking.

Transcriptomic techniques facilitate the comprehensive evaluation of changes at the transcriptional level to elucidate the underlying mechanisms of many antimicrobial agents [11, 12]. However, transcriptomic techniques often fail to capture alterations in post-transcriptional regulation. Metabolomic analysis complements transcriptomics by identifying final changes induced by external stimuli in organisms.

Previous studies have demonstrated that dietary supplementation with gallnut tannins effectively mitigates the adverse effects of necrotic enteritis in broilers [13, 14]. Since gallnut tannins are primarily composed of 5–12 galloyl-glucose units, this study selected pentagalloylglucose (PGG) and tannic acid (TA) for mechanistic investigations based on these findings. Their structural details are illustrated in Supplementary Fig. 1. This study investigated the multi-target interactions of PGG and TA against *C. perfringens* at the transcriptional and metabolic levels, and defined their antimicrobial mechanisms. Collectively, the findings of this study map the cascading effects of tannins on bacterial survival, virulence, and host interactions to inform the development of plant polyphenol-based strategies against *C. perfringens.*

## Materials and methods

### Materials

PGG (CAS No. 14937-32-7, purity above 98%) was procured from Chengdu Angsaisi Biotechnology Co., Ltd. (Chengdu, China), while TA (CAS No. 1401-55-4, purity above 98%) was obtained from Shanghai Macklin Biochemistry Science and Technology Co., Ltd. (Shanghai, China). Fluid thioglycollate medium (FTG, GCM801) was acquired from Beijing Land Bridge Technology Co., Ltd. (Beijing, China). Penicillin/streptomycin (V900929) was acquired from Merck (Shanghai, China). Dulbecco’s modified eagle medium (DMEM)-F12 (sh30023.01) was sourced from Cytiva Hyclone (Logan, UT, USA). *C. perfringens* (type A CVCC52) was purchased from the China Veterinary Culture Collection Center.

### Inhibitory effects of PGG and TA against *C. perfringens*

*C. perfringens* was anaerobically incubated until it reached the logarithmic phase. The bacterial culture medium was modified to achieve an optical density of 1 × 10^7^ colony-forming units (CFU)/mL. PGG or TA was added to a final 0.125 mg/mL concentration. The mixture was incubated with *C. perfringens* for 6 h. The supernatant was passed through a 0.22-µm filter membrane. The potassium (K^+^) and magnesium (Mg^2+^) ion content was quantified using an inductively coupled plasma mass spectrometer (Agilent 7800, Agilent, Inc., USA), and the internal standard method was used for quantification.

Furthermore, *C. perfringens* was anaerobically cultured to the logarithmic phase (2 × 10^8^ CFU/mL). The culture medium was centrifuged at 5,000 × *g* for 15 min, and the supernatant was collected. The bacteria were washed thrice with sterile phosphate-buffered saline (PBS). The treatment group was treated with 0.125 mg/mL PGG or TA. Subsequently, an equal volume of PBS was administered to the control group. The bacteria were incubated anaerobically for 90 min, centrifuged at 5,000 × *g* for 15 min, and washed thrice using PBS. The bacterial sediment was reconstituted in PBS and stained with SYTO (KGA260, Shanghai Solely Biotechnology Co., Ltd., Shanghai, China) and propidium iodide (PI, C0080, Beijing Solarbio Biotechnology Co., Ltd., Beijing, China). The integrity of the bacterial membrane was analyzed using a flow cytometer (BD FACSCanto II, USA).

### Transmission electron microscopic observation of bacterial microstructure

*C. perfringens* was cultured anaerobically to the logarithmic phase. The culture medium underwent centrifugation at 5,000 × *g* for 15 min. After discarding the supernatant, the bacteria were washed thrice using sterile PBS. The OD_600_ of the medium was adjusted to 0.2 with PBS. The bacteria were treated with 0.125 mg/mL PGG or TA for 3 h and fixed with an electron microscope fixative for 12 h. The fixed samples were rinsed thrice with PBS, followed by sequential dehydration in ethanol. Dehydration was completed with acetone and epoxy penetration. The microstructure of *C. perfringens* was observed and photographed using a transmission electron microscope (TECNAI G 20 TWIN, FEI Company, USA).

### Transcriptomic analysis

*C. perfringens* was cultured anaerobically to the logarithmic phase. The culture medium was centrifuged at 5,000 × *g* for 15 min. The supernatant was removed, and the bacteria were washed thrice with sterile PBS. The OD_600_ of the medium was adjusted to 0.2 with PBS. PGG or TA (0.125 mg/mL) was added to the culture and incubated for 3 h before bacterial cell lysis. Bacterial RNA was extracted and reverse-transcribed into cDNA. cDNA sequencing was performed on the Illumina platform using synthetic technology. The raw data were filtered and aligned to the *C. perfringens* reference genome. Based on the alignment results, the expression of genes was quantified and visualized. Differential expression of the genes was analyzed using DESeq, with differentially expressed genes (DEGs) identified based on the criteria of |log_2_ fold change (FC)| > 1 and *P* < 0.05. All treatments were conducted with three biological replicates.

### Metabolomic analysis

Metabolites were extracted and analyzed per the protocol described by Yu et al. [15]. Detection analysis was performed using electrospray ionization. The raw data underwent processing with MSDIAL software. Positive and negative ion data were normalized separately, and the integrated ion peaks were analyzed using Python software for pattern recognition. Data preprocessing was performed using unit variance scaling. All treatments were conducted with six biological replicates.

### Determination of α toxin levels in the supernatant

Bacterial cultures in the logarithmic phase were centrifuged at 4,000 × *g* for 20 min. The supernatant was gathered and co-cultured with 0.125 mg/mL PGG or TA for 2 h. The α toxin content was measured using a *C. perfringens* α toxin enzyme-linked immunosorbent assay (ELISA) kit (F210162-A, Shanghai Kexing Trading Co., Ltd., Shanghai, China).

### Molecular docking

The molecular binding affinity of the small molecules PGG and TA with *C. perfringens* α toxin protein targets was investigated using the molecular docking method. The CPA protein crystal structure (PDB ID: 2 WXT) was downloaded from the RCSB Protein Data Bank (https://www.rcsb.org/), and the SDF files of the two-dimensional structures of the small molecules were downloaded from the PubChem database (https://pubchem.ncbi.nlm.nih.gov/). Potential active sites in the protein structures were predicted using the DEEPSITE module on the PlayMolecule website (https://playmolecule.com/) and the PROTEINS PLUS website (https://proteins.plus/). The predicted active sites were entered into the Grid Box module of AutoDock Tools, with the coordinates of the most confident active site set at (*x* = −1.89, *y* = 66.41, *z* = 39.98) and the Grid Box size set to 20*x*, 20*y*, 20*z*. Molecular docking was performed using AutoDock Vina 1.1.2 software. The protein-ligand structures with optimal binding energy were exported as PDB files. The three-dimensional and two-dimensional docking results were visualized using PyMol 2.5 and Schrodinger software, respectively.

### Cell culture

#### Determination of cell viability

Intestinal epithelial cells were isolated from chicken embryos during 18 d of incubation. *C. perfringens* was diluted to 10^5^ CFU/mL with DMEM/F12 without Penicillin–streptomycin. The experiment included six treatment groups: negative control; positive control (with *C. perfringens*); TA (7.8 µg/mL TA); PGG (7.8 µg/mL PGG); PTA (*C. perfringens* + 7.8 µg/mL TA); and PPGG (*C. perfringens* + 7.8 µg/mL PGG). All treatment groups were incubated for 6 h in a 37 °C, 5% CO_2_ incubator.

Cell cytotoxicity was assessed using the Calcein/PI Cell Activity and Cytotoxicity Assay Kit (C2015M, Beyotime Biotechnology Co., Ltd., Shanghai, China), according to the manufacturer’s instructions. The SpectraMax i3x Multi-Mode Detection Platform (Ex/Em: 494/517 nm, Ex/Em: 535/617 nm) was used to read fluorescence values.

After culturing, the lactate dehydrogenase content of supernatants was assayed using the Lactate Dehydrogenase Cytotoxicity Assay Kit (C0016, Beyotime Biotechnology Co., Ltd., Shanghai, China) following the manufacturer’s instructions.

#### Detection of α toxin content in the cell supernatants

The treated cell supernatant was collected and centrifuged at 3,000 × *g* for 10 min, and α toxin content was quantified using the *C. perfringens* α toxin ELISA kit (F210162-A, Shanghai Kexing Trading Co., Ltd., Shanghai, China) according to the manufacturer’s instructions.

#### Detection of intestinal barrier function

The upper chamber of a Transwell insert in a 12-well plate was filled with 300 µL of DMEM/F12, and the lower chamber was filled with 800 µL of DMEM/F12. Trans-epithelial electrical resistance (TEER) was measured using an EVOM2 Transendothelial/Epithelial Electrical Resistance System (MW09-EVOM2, World Precision Instruments, USA). Before measurement, probes were sterilized and equilibrated in DMEM/F12 for 15 min. Three stable readings were made at three randomly selected sites per well.

To assess paracellular permeability, intestinal epithelial cells were cultivated in the upper chamber of a Transwell insert in a 12-well plate. To assess barrier function, 250 µL of DMEM/F12 and 250 µL of 1 mg/mL fluorescein isothiocyanate (FITC) solution (4 kDa) were added to the upper chamber, while 500 µL of DMEM/F12 was added to the lower chamber. The cells were incubated at 37 °C in a 5% CO_2_ incubator for 2 h. Subsequently, 100 µL of medium from the lower chamber was transferred to a black 96-well plate. The fluorescence intensity was measured using a SpectraMax i3x Multi-Mode Detection Platform (Molecular Devices, LLC, CA, USA). FITC values were quantified against a calibration curve for absolute quantification.

#### Expression of intestinal inflammation-related genes

Cells were harvested from different treatment groups using 6-well plates. To extract total RNA, 1 mL of TRIzol reagent (Invitrogen Life Technologies, Carlsbad, USA) was added to each plate. The extracted RNA was reverse-transcribed using the M-MLV cDNA synthesis kit (Invitrogen Life Technologies). Reverse transcription was carried out in a 7500 Fluorescence Detection System (Applied Biosystems, Foster City, CA, USA) using the SYBR^®^ Select Master Mix PCR kit (Takara Biotechnology Co., Ltd., Beijing, China). β-Actin served as the internal reference gene for normalization. Quantitative results were analyzed statistically following the method outlined by Fu et al. [16]. The primer sequences for all genes are presented in Table 1.

**Table 1:** Sequences of primers used for gene quantification in vitro

Gene name	Primer sequence (5′→3′)	NCBI serial number
*β-actin*	F-CAACACAGTGCTGTCTGGTGGTAC	NM_205518.1
	R-CTCCTGCTTGCTGATCCACATCTG	
*IL-10*	F- CGCTGTCACCGCTTCTTCA	NM_001004414.4
	R-TCCCGTTCTCATCCATCTTCTC	
*TGF-β*	F- GCCGACACGCAGTACACCAAG	NM_001318456.1
	R- GCAGGCACGGACCACCATATTG	
*IL-18*	F- GTGTGTGCAGTACGGCTTAG	NM_204608.1
	R- TCCACTGCCAGATTTCACCT	
*TNF-α*	F-CCCCTACCCTGTCCCACAA	NM_204267
	R-TGAGTACTGCGGAGGGTTCAT	
*IFN-γ*	F-CTCGCAACCTTCACCTCACCATC	NM_205149.1
	R-CAGGAACCAGGCACGAGCTTG	

*F* Forward primer, *R* Reverse primer, primers were synthesized at Shanghai Sangon Biotech Co., Ltd.

### In vivo experiment

#### Experimental design

Male C57BL/6 mice (6–8 weeks old; *n* = 84) were randomly divided into seven weight-matched groups (*n* = 12 mice/group): negative control (NC, untreated), positive control (PC, *C. perfringens* challenge only), clindamycin-treated with challenge (CLI, 50 mg/kg clindamycin), low-dose TA with challenge (TAL: 100 mg/kg), high-dose TA with challenge (TAH: 200 mg/kg), low-dose PGG with challenge (PGGL: 100 mg/kg), and high-dose PGG with challenge (PGGH: 200 mg/kg).

#### Construction of a necrotic enteritis model

Following a 7-d acclimation period, the *C. perfringens* infection model was established as previously described [17] with modifications. Mice received antibiotic water (1 g/L amoxicillin, 0.5 g/L vancomycin, 1 g/L neomycin, and 1 g/L metronidazole) for 7 d to suppress gut microbiota. Following a 2-d recovery period, mice were intraperitoneally injected with 30 mg/kg clindamycin. Subsequently, daily oral gavage of PGG, TA, or vehicle was initiated alongside a 150 μL challenge of *C. perfringens* CVCC52 suspension (3 × 10^9^ CFU/mL) for three consecutive days.

#### Sample collection

Six mice per group were euthanized via cervical dislocation 3 d post-challenge. The abdominal cavity was dissected, the intestines were carefully separated, and a 1-cm length of the mid-ileum was collected and fixed in paraformaldehyde. Additionally, molecular samples of the ilea were prepared and stored in liquid nitrogen.

#### Intestinal morphological indices

The mid-ileum tissues, preserved in 4% paraformaldehyde, were dehydrated, embedded in paraffin, and sliced into 5-µm-thick sections. Tissue sections underwent histological staining using hematoxylin and eosin for morphological assessment. Morphometric analysis of ileal architecture was performed according to the methodology described by Frankel et al. [18].

#### Quantitative reverse transcription-PCR (qRT-PCR) analysis

Total RNA was isolated from 100-mg tissue samples using TRIzol reagent (Invitrogen) following the manufacturer’s protocol. Reverse transcription was performed using a cDNA synthesis kit (Takara Biotechnology Co., Ltd., Beijing, China). qRT-PCR assays were conducted on an Applied Biosystems 7500 Fast System using the SYBR® Select Master Mix PCR kit (Takara Biotechnology Co., Ltd., Beijing, China), under standardized cycling conditions. Gene expression levels were normalized to glyceraldehyde-3-phosphate dehydrogenase (*GAPDH*) and calculated using the 2^−ΔΔCt^ method [16]. Primer sequences are listed in Table 2.

**Table 2:** Sequences of primers used for gene quantification in vivo

Gene name	Primer sequence (5′→3′)	NCBI serial number
*GAPDH*	F-ATGGTGAAGGTCGGTGTGAA	NM_008084.4
	R-TGGAAGATGGTGATGGGCTT	
*CCL3*	F-TTCTCTGTACCATGACACTCTGC	NM_011337.2
	R-CGTGGAATCTTCCGGCTGTAG	
*CCL4*	F-TTCCTGCTGTTTCTCTTACACCT	NM_013652.2
	R-CTGTCTGCCTCTTTTGGTCAG	
*CCL5*	F-GCTGCTTTGCCTACCTCTCC	NM_013653.3
	R-TCGAGTGACAAACACGACTGC	
*CXCL1*	F-TGCACCCAAACCGAAGTCAT	NM_008176.3
	R-ACTTGGGGACACCTTTTAGCA	
*CXCL2*	F-CCAACCACCAGGCTACAGG	NM_009140.2
	R- GCGTCACACTCAAGCTCTG	
*CXCL5*	F-TGCCCTACGGTGGAAGTCATA	NM_009141.3
	R-TGCATTCCGCTTAGCTTTCTTT	
*IL-10*	F-GCTCTTACTGACTGGCATGAG	NM_010548.2
	R-CGCAGCTCTAGGAGCATGTG	
*IL-22*	F-ATGAGTTTTTCCCTTATGGGGAC	NM_016971.2
	R-GCTGGAAGTTGGACACCTCAA	
*TNF-a*	F-GTTGTACCTTGTCTACTCCCAG	NM_001278601.1
	R-GGTTGACTTTCTCCTGGTATGAG	
*MUC2*	F-ATTCCCAAAGTCCAGCTCGC	NM_023566.4
	R-CAAGCTCCGGCATCATTCGT	
*OCLD*	F-TCGGTACAGCAGCAATGGT	NM_008756.2
	R-GTTGATCTGAAGTGATAGGTGGAT	
*JAM-A*	F-AGCAGCAATTAGCAAGATAGGT	NM_172647.2
	R-AACAGCCGAGTTGGTTGAAG	

*F* Forward primer, *R* Reverse primer, primers were synthesized at Shanghai Sangon Biotech Co., Ltd.

### Statistical analysis

SPSS 26.0 (SPSS, Inc., Chicago, IL, USA) was used to perform the data analysis. The significance was determined using a one-way ANOVA with Dunnet’s multiple comparison post-hoc test for data. When there are only two groups of data in the results, use the independent samples *t*-test for analysis. A *P* < 0.05 was considered to indicate statistical significance, and *P* < 0.01 was considered to indicate statistical highly significance.

## Results

### Effects of PGG and TA on *C. perfringens* proliferation

**Fig. 1:**
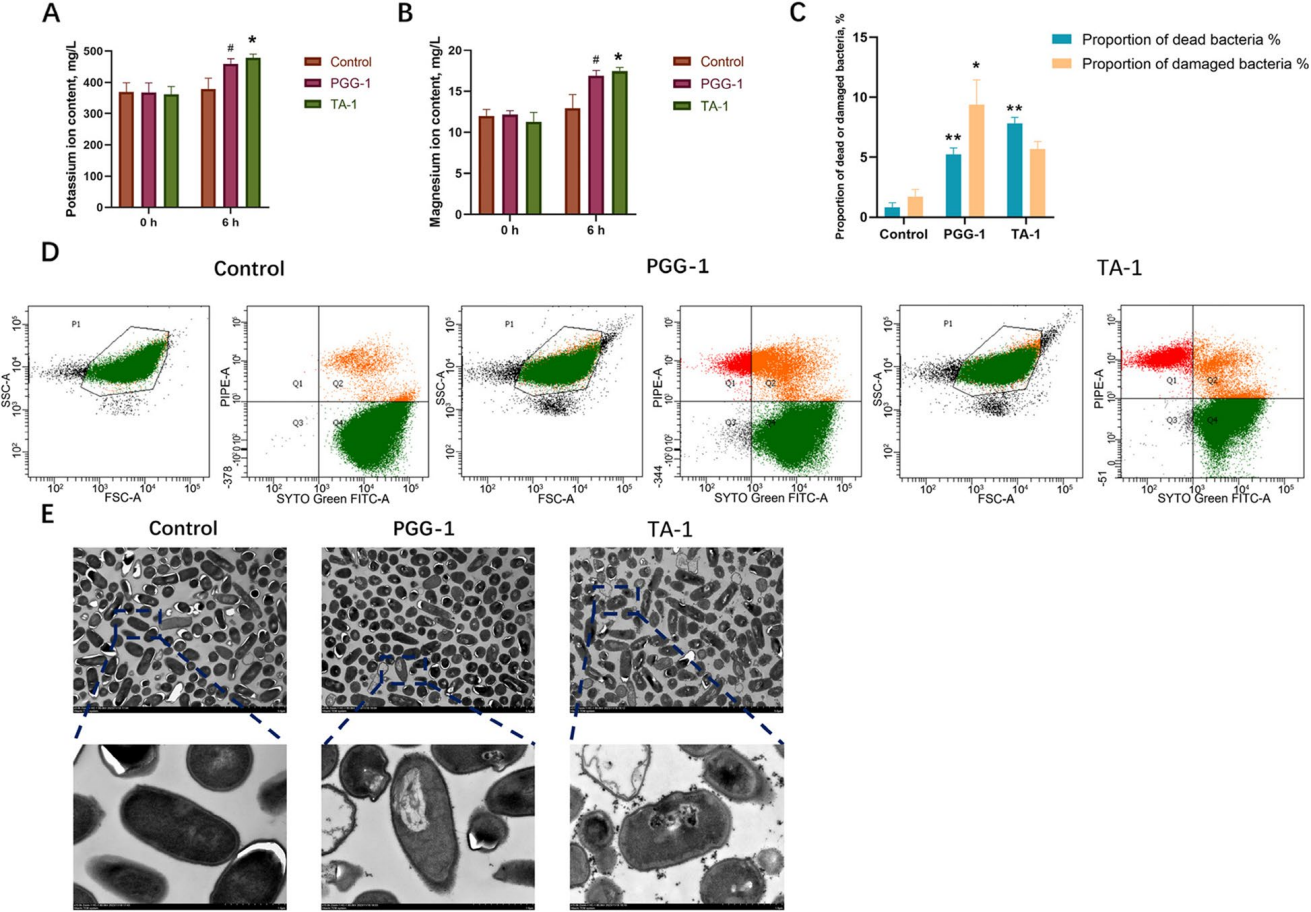
Inhibitory effects of PGG and TA on *Clostridium perfringens*. **A** and **B** Effects of PGG and TA on the potassium and magnesium ion contents in the bacterial supernatant, respectively. **C** and **D** Effects of PGG and TA on the percentage of damage and death of *C. perfringens* cells, as determined through flow cytometry analysis. **E** Effects of PGG and TA on the microstructure of *C. perfringens* as observed by transmission electron microscopy. Control: *C. perfringens* group; PGG-1: 0.125 mg/mL PGG treatment + *C. perfringens* group; TA-1: 0.125 mg/mL TA treatment + *C. perfringens* group. ^#^0.05 ≤ *P* < 0.1; ^*^P < 0.05;^**^P < 0.01; PGG, pentagalloylglucose; TA, tannic acid. *n* = 3

The K^+^ and Mg^2+^ content of the bacterial supernatants was determined using inductively coupled plasma mass spectrometry (Fig. 1A and B). After 6 h of treatment, compared with the control group, the PGG-1 group tended to increase the contents of K^+^ and Mg^2+^ in the supernatant (*P* < 0.1). The TA-1 groups had significantly higher K^+^ and Mg^2+^ concentrations in the bacterial supernatant (*P* < 0.05) than the control group. Additionally, the PGG-1 and TA-1 groups exhibited significantly higher proportions of bacterial death than the control group (*P* < 0.01; Fig. 1C and D). Specifically, PGG was responsible for 9.4% of bacterial damage and 5.2% of bacterial death, while TA caused 5.7% of bacterial damage and 7.8% of bacterial death. Thus, TA had a stronger cytotoxic effect while PGG caused damage to a larger proportion of cells.

TEM was employed to detect the ultrastructural changes in *C. perfringens* after treatment with PGG and TA. In the control group, the bacteria appeared rod-shaped with smooth surfaces, clear and intact cell membrane structures, and full, dense contents (Fig. 1E). In contrast, the bacterial cells treated with PGG or TA had an irregular shape, with some appearing hollow, with leaking contents and ruptured cell membranes. The TA-1 group exhibited a more severe degree of destruction than the PGG-1 group.

### Effects of PGG and TA on *C. perfringens* transcriptome

**Fig. 2:**
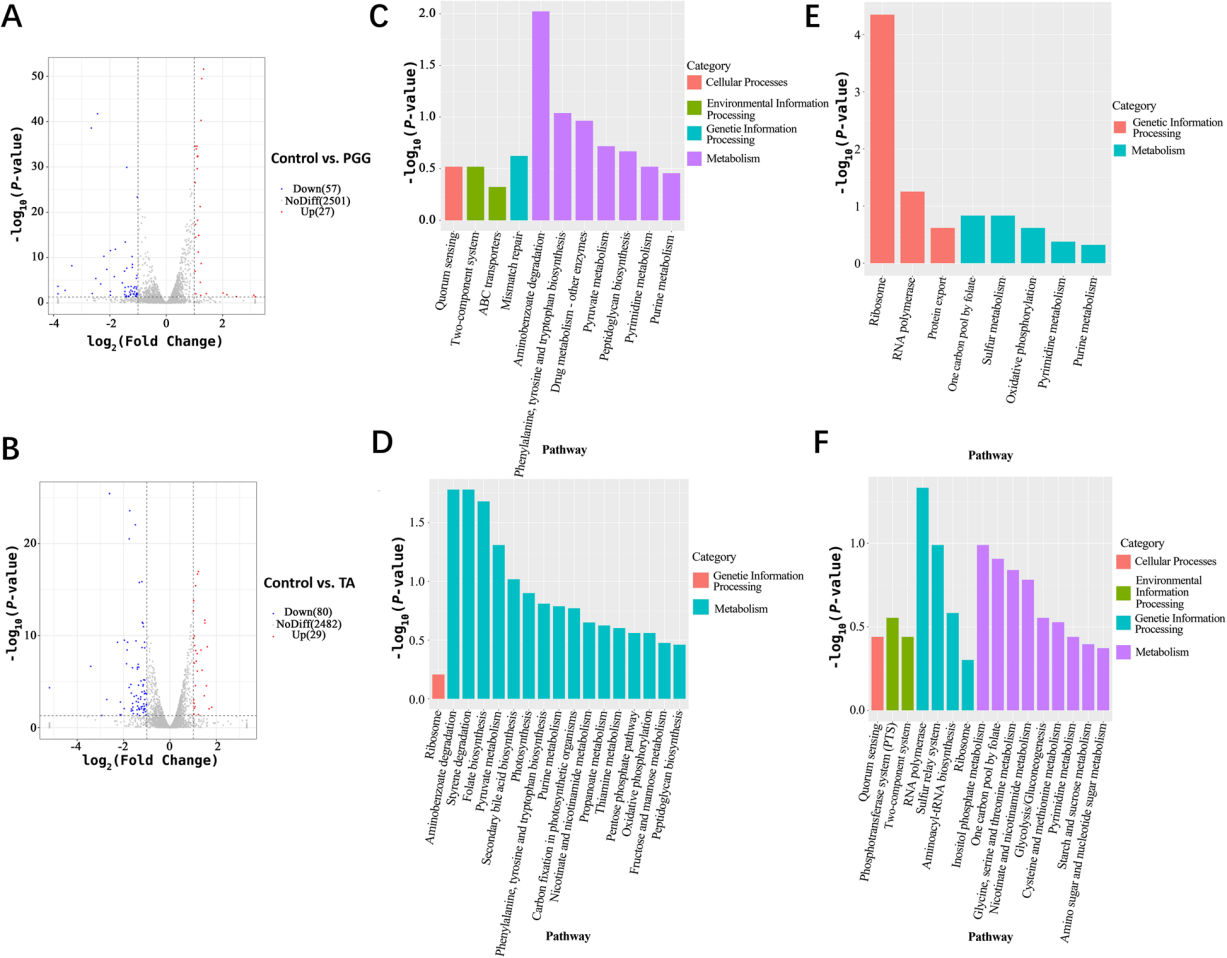
Transcriptomic analysis of the effects of PGG and TA on *Clostridium perfringens*. **A** and ** B** Volcano plots of differentially expressed genes following PGG and TA treatments. **C** and **E** Downregulated and upregulated pathways identified via KEGG pathway analysis of the differentially expressed genes following PGG treatment. **D** and **F** Downregulated and upregulated pathways identified via KEGG pathway analysis of the differentially expressed genes following TA treatment. Control: *C. perfringens* group; PGG: 0.125 mg/mL of PGG treatment + *C. perfringens*; TA: 0.125 mg/mL of TA treatment + *C. perfringens*. KEGG, Kyoto Encyclopedia of Genes and Genome. *n* = 3

To pinpoint the key DEGs implicated in the proliferation of *C. perfringens* influenced by PGG and TA, transcriptomic analysis was conducted on the control, PGG, and TA groups. Compared with those in the control group, 27 genes were upregulated in the PGG group, including membrane protein insertion efficiency factors, *yidD*, *asrB*, and *rpsD*; and ribosomal protein-related genes, *rplE*, *rplB*, and *rpmC* (Fig. 2A). Additionally, 57 genes were downregulated, including the transcriptional regulators *nrdR*, *mscL*, *splB*, *xseA*, and *aroF*, which have reparative effects on injury. In the TA group, 29 genes were upregulated, including the ribosomal protein-related genes, *rplB* and *mobB*; the transcriptional elongation factors, *greA* and *thyX*; ethanolamine-utilizing microcompartment protein, *EutM*; and the spore-forming integral membrane protein, *ylbJ* (Fig. 2B). Additionally, 80 genes were downregulated, including *mscL*; the transcriptional regulators, *nrdR*, *ylqF*, and *folE*; the flagellin-associated gene, *fliB*; the damage repair-related gene, *ric*; and the ribosome-associated genes, *rpiB* and *rsgA*. Further transcriptomic analysis revealed that *CPA*, a gene encoding α toxin, was significantly downregulated in the PGG and TA groups, and *CPB2*, a gene encoding β2 toxin, was significantly downregulated in the PGG group (Supplementary Fig. 2).

To investigate the function of these non-redundant DEGs in bacterial biological processes, functional analyses were performed using the Kyoto Encyclopedia of Genes and Genome (KEGG) pathway database. PGG downregulated pathways related to quorum sensing, mismatch repair, amino acid biosynthesis, and peptidoglycan biosynthesis (Fig. 2C). Conversely, PGG upregulated pathways associated with ribosomal function, pyrimidine and purine metabolism, and RNA polymerase activity (Fig. 2E). TA-regulated genes were predominantly associated with metabolism, with significant downregulation in pyruvate metabolism, purine metabolism, peptidoglycan biosynthesis, glucose metabolism, and amino acid synthesis-related pathways (Fig. 2D). In contrast, TA significantly upregulated pathways related to quorum sensing, the phosphotransferase system, and pyrimidine metabolism (Fig. 2F). Hence, the common mechanisms by which PGG and TA inhibit *C. perfringens* involve interference with biosynthesis processes related to quorum sensing, the two-component system, oxidative phosphorylation, pyruvate metabolism, and peptidoglycan biosynthesis. The differences between the PGG and TA transcriptomes are detailed in Supplementary Fig. 3. Compared with the TA treatment group, PGG appears more inhibitory to bacterial behavior, perception of the external environment, and signaling. Conversely, TA shows a greater inhibitory effect on bacterial metabolism and oxidative phosphorylation.

### Effects of PGG and TA on the *C. perfringens* metabolome

**Fig. 3:**
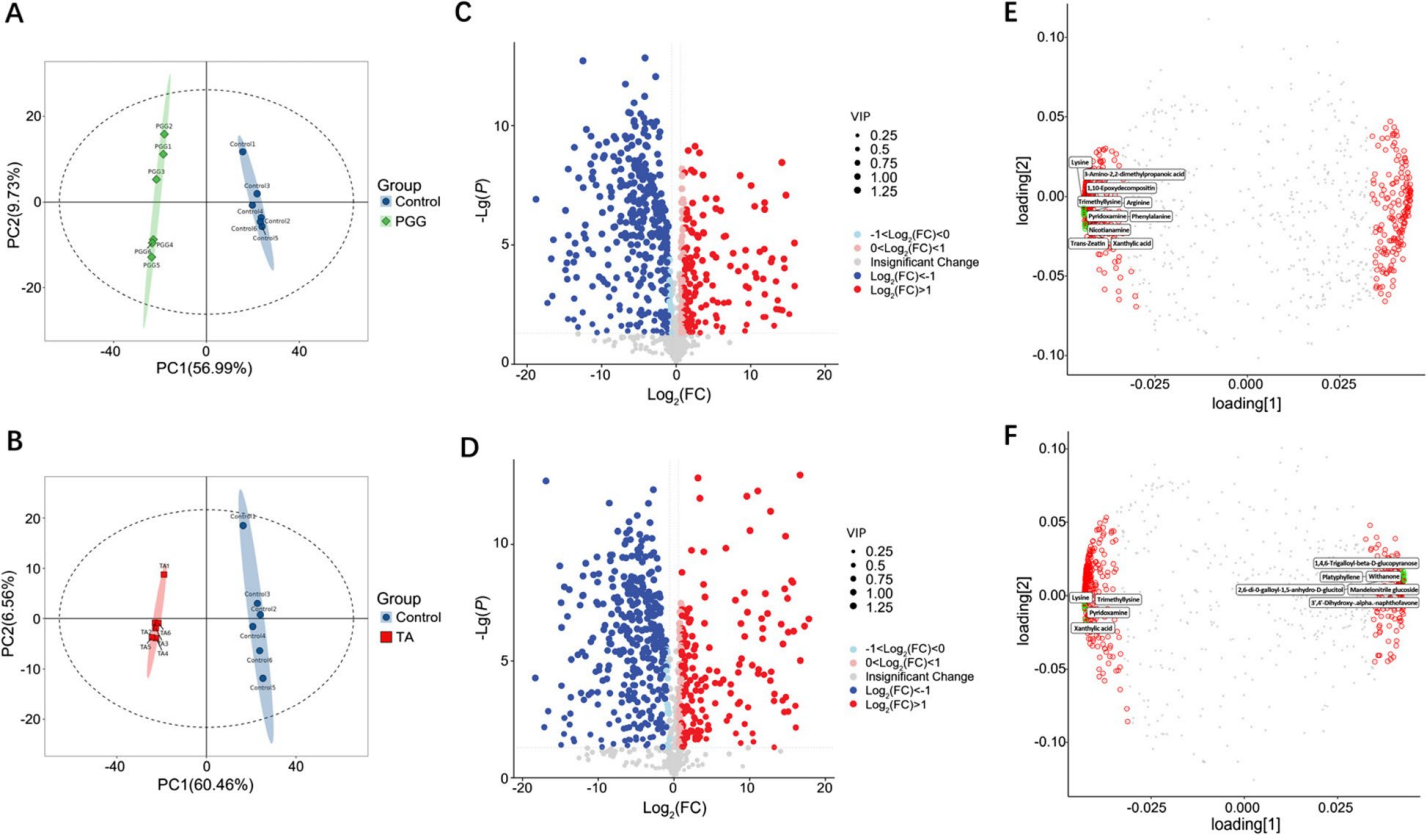
Metabolomic analysis of the effects of PGG and TA on *Clostridium perfringens*. **A** and **B** PCA plots of metabolites after PGG and TA treatments. **C** and ** D** Volcano plots of differential metabolites after PGG and TA treatments, respectively; Red dots: upregulated metabolites, blue dots: downregulated metabolites. **E** and **F** PLS-DA loading plots of PGG and TA, respectively. The top 10 VIP values are marked with green circles, and the remaining metabolites with VIP values > 1 are indicated with red circles. Control: *C. perfringens* group; PGG: 0.125 mg/mL of PGG treatment + *C. perfringens*; TA: 0.125 mg/mL of TA treatment + *C. perfringens*. PCA, principal component analysis; PLS-DA, partial least squares discriminant analysis. *n* = 6

Considering that the effects of PGG and TA on *C. perfringens* were primarily related to metabolism, the metabolomic profiles of *C. perfringens* treated with PGG or TA were analyzed (Fig. 3). The principal component analysis (PCA) of samples treated with PGG or TA showed complete separation from the control group (Fig. 3A and B), indicating that PGG and TA cause significant changes in the metabolite profiles of *C. perfringens*. Compared with those in the control group, 403 metabolites exhibited significant differences after PGG treatment (Fig. 3C); the primary differential metabolites were arginine, lysine, phenylalanine, trimethyllysine, xanthine nucleotides, 3-amino-2,2-dimethylpropanoic acid, and 1,10-epoxydecompositin (Fig. 3E). Similarly, 431 metabolites were differentially abundant following TA treatment (Fig. 3D), with the main metabolites being lysine, trimethyllysine, xanthine nucleotides, pyridoxamine, 1,4,6-trigalloyl-beta-D-glucopyranose, 2,6-di-O-galloyl-1,5-anhydro-D-glucitol, and 3′,4′-dihydroxy-alpha-naphthoflavone (Fig. 3F).

**Fig. 4:**
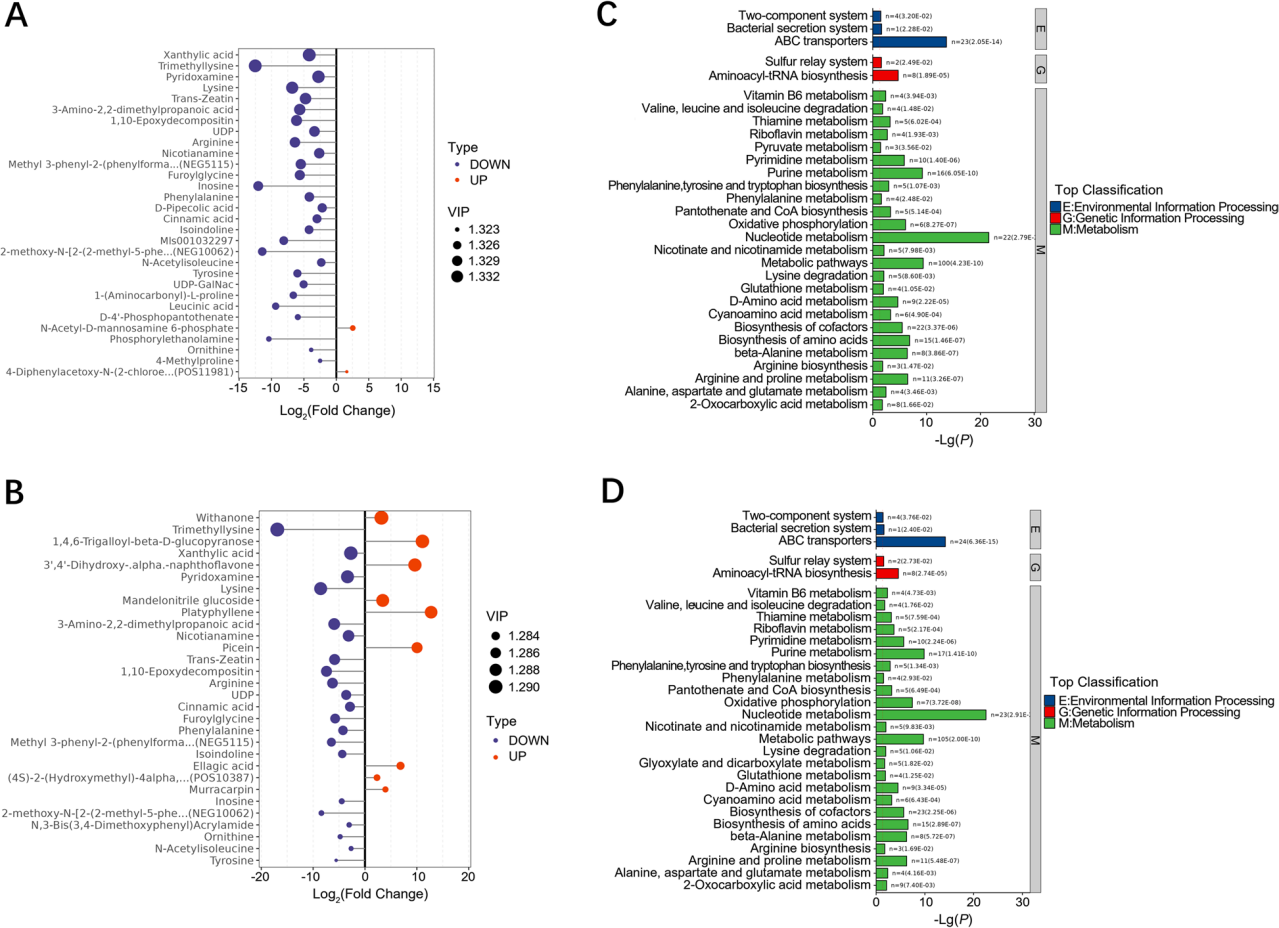
Analysis of differential metabolites after PGG and TA treatment of *Clostridium perfringens*. **A** and ** B** Top 30 upregulated and downregulated metabolites following PGG and TA treatments; dot size corresponds to the VIP values obtained from the OPLS-DA: larger dots indicate higher VIP values; Red dots: upregulated metabolites, blue dots: downregulated metabolites. **C** and **D** KEGG pathway analyses of differential *C. perfringens* metabolites after PGG and TA treatments. PGG, pentagalloylglucose; TA, tannic acid; VIP, variable importance in projection; OPLS-DA, orthogonal partial least squares- differential analysis. KEGG, Kyoto Encyclopedia of Genes and Genome. *n* = 6

The top 30 differentially important metabolites were identified (Fig. 4A and B). In comparison to those in the control group, metabolites downregulated after PGG treatment primarily included amino acids crucial for cell activities, including lysine, tyrosine, arginine, and ornithine, along with isoindole and phosphatidylethanolamine, among others (Fig. 4A). Meanwhile, the differentially upregulated metabolites included N-acetyl-D-mannosamine 6-phosphate and 4-diphenylacetoxy-N-(2-chloroethyl) piperidine hydrochloride. TA treatment upregulated withanone, 1,4,6-trigalloyl-beta-D-glucopyranose, mandelonitrile glucoside, platyphyllene, picein, and murracarpin, while downregulating lysine, arginine, phenylalanine, ornithine, and tyrosine (Fig. 4B).

**Fig. 5:**
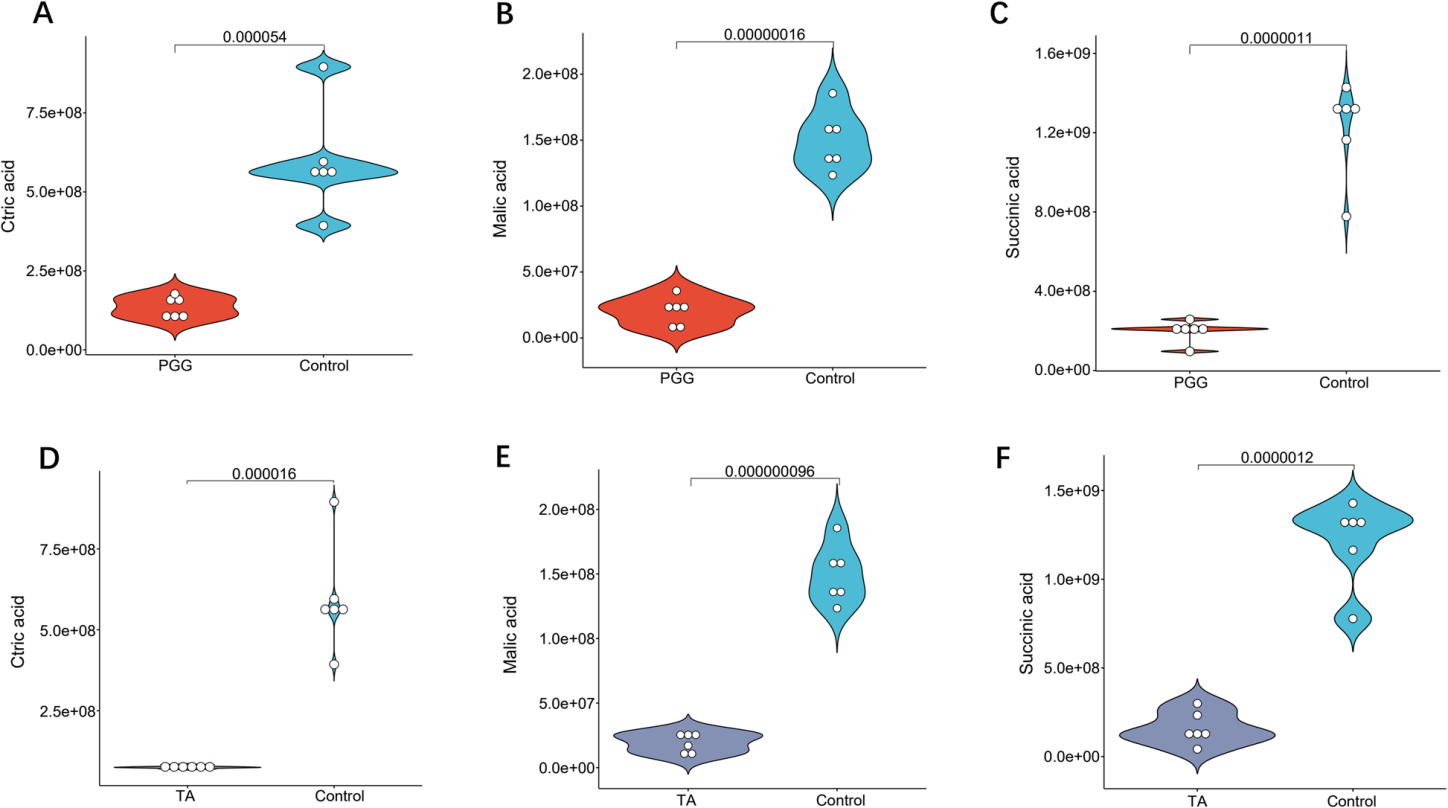
Effects of PGG and TA on  energy-related metabolites in the *Clostridium perfringens*. **A–C** Effects of PGG on citric acid, malic acid, and succinic acid contents. **D–F** Effects of TA on citric acid, malic acid, and succinic acid contents. Control: *C. perfringens* group; PGG: 0.125 mg/mL of PGG treatment + *C. perfringens*; TA: 0.125 mg/mL of TA treatment + *C. perfringens*. *n* = 6

KEGG analysis of the metabolites revealed that the primary affected pathways by PGG and TA included the two-component system, bacterial secretion system, ABC transporter system, sulfur transporter system, and biosynthesis of tRNAs that bind amino acids (Fig. 4C and D). Moreover, PGG and TA significantly interfered with various metabolic pathways crucial for life activities, encompassing amino acid biosynthesis and metabolism, vitamin synthesis and metabolism, as well as purine and pyrimidine metabolism. Further analysis of energy metabolism-related differentials revealed that the levels of citric, succinic, and malic acid were markedly reduced by PGG and TA treatment (Fig. 5A–F).

### Combined transcriptomics and metabolomics analysis of the effects of PGG and TA on *C. perfringens*

**Fig. 6:**
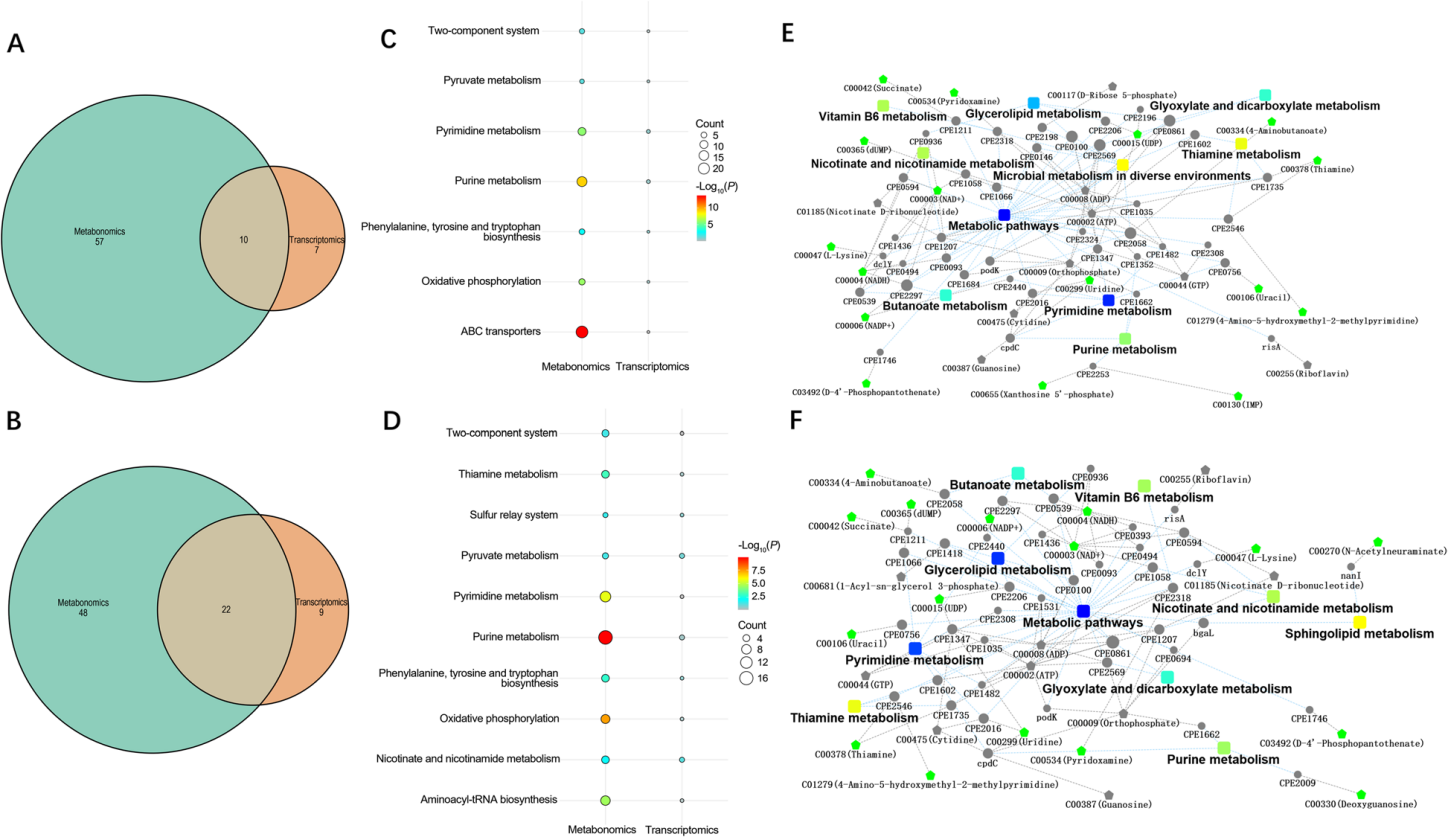
Combined transcriptomic and metabolomic analysis of the effects of PGG and TA on *Clostridium perfringens.* **A** and **B** Venn diagrams of the shared pathways in the transcriptome and metabolome for PGG and TA treatments, respectively. **C** and **D** Shared pathways after treatments with PGG and TA, respectively. **E** and **F** Network diagrams mapping differential metabolites and differential genes after treatments with PGG and TA, respectively; Rectangular nodes: KEGG pathways; significant *P*-values indicated by a yellow–blue gradient (yellow: smaller *P*-values); Circular nodes: genes; pentagons: metabolites (red: upregulation, green: downregulation). PGG, pentagalloylglucose; TA, tannic acid; KEGG, Kyoto Encyclopedia of Genes and Genomes

To gain a thorough understanding of how PGG and TA affect *C. perfringens* proliferation, a transcriptome–metabolome interaction network was generated for DEGs and differentially abundant metabolites (Fig. 6A and B). KEGG enrichment analysis identified 10 pathways in the PGG group that intersected the transcriptomic and metabolomic data, including amino acid biosynthesis, oxidative phosphorylation, ABC transporter system, two-component system, pyruvate metabolism, and purine metabolism (Fig. 6C). In the TA group, 22 pathways were identified, including those affected by PGG as well as thiamine metabolism, the sulfur transport system, and the biosynthesis of tRNAs bound to amino acids (Fig. 6D).

This combined analysis also identified the pathways through which PGG and TA may inhibit *C. perfringens* proliferation. The network analysis revealed that PGG affects the metabolism of glycerol ester, glyoxylate, dicarboxylate niacin, nicotinamide, thiamine, butyric acid, purine, pyrimidine, and vitamin B_6_ (Fig. 6E). In addition to these pathways, TA affects sphingolipid metabolism (Fig. 6F). Overall, this combined analysis successfully identified the relevant pathways through which PGG and TA inhibit *C. perfringens* proliferation.

### Effects of PGG and TA on α toxin content in bacterial supernatants and molecular docking

**Fig. 7:**
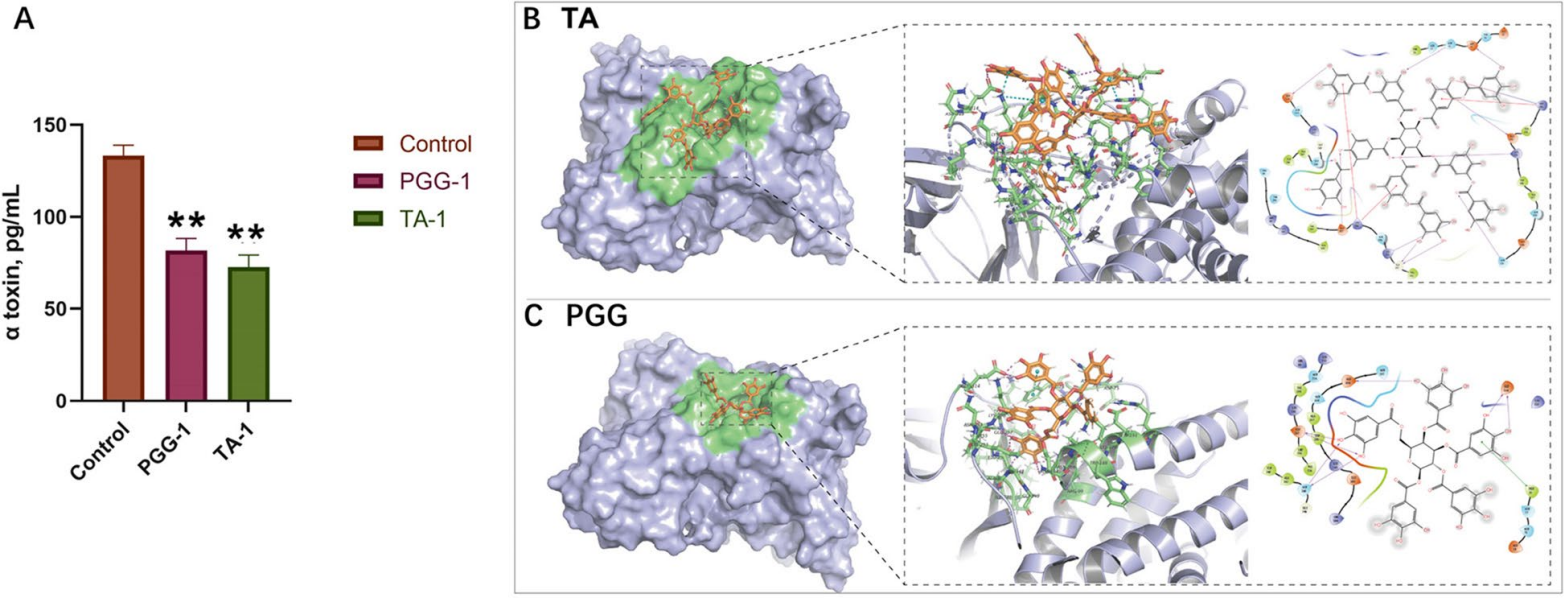
Effects of PGG and TA on α toxin produced by *Clostridium perfringens*. **A** Effects of PGG and TA on α toxin in the supernatant of *C. perfringens*, *n* = 6. **B** and **C** Schematic diagrams of molecular docking of TA and PGG with α toxin, respectively. Control: *C. perfringens* group; PGG-1: 0.125 mg/mL of PGG treatment + *C. perfringens*; TA-1: 0.125 mg/mL of TA treatment + *C. perfringens*. ^**^P < 0.01; PGG, pentagalloylglucose; TA, tannic acid

The α toxin levels in the supernatant following culture with PGG and TA were markedly decreased compared with those in the control (*P* < 0.01; Fig. 7A). Molecular docking results indicated that PGG and TA interact with α toxin primarily through hydrogen bonds, π-cation interactions, π–π interactions, and hydrophobic interactions (Fig. 7B and C). The docking score of PGG with α toxin was 6.867 kcal/mol, whereas that of TA with α toxin was 8.525 kcal/mol (Table 3).

**Table 3:** Docking scores for PGG and TA with α toxin

PDB ID	Ligand	Docking score, kcal/mol
2 WXT	PGG	−6.867
	TA	−8.525

*PGG* Pentagalloylglucose, *TA* Tannic acid

### Protective effects of PGG and TA on *C. perfringens*-infected intestinal epithelial cells

**Fig. 8:**
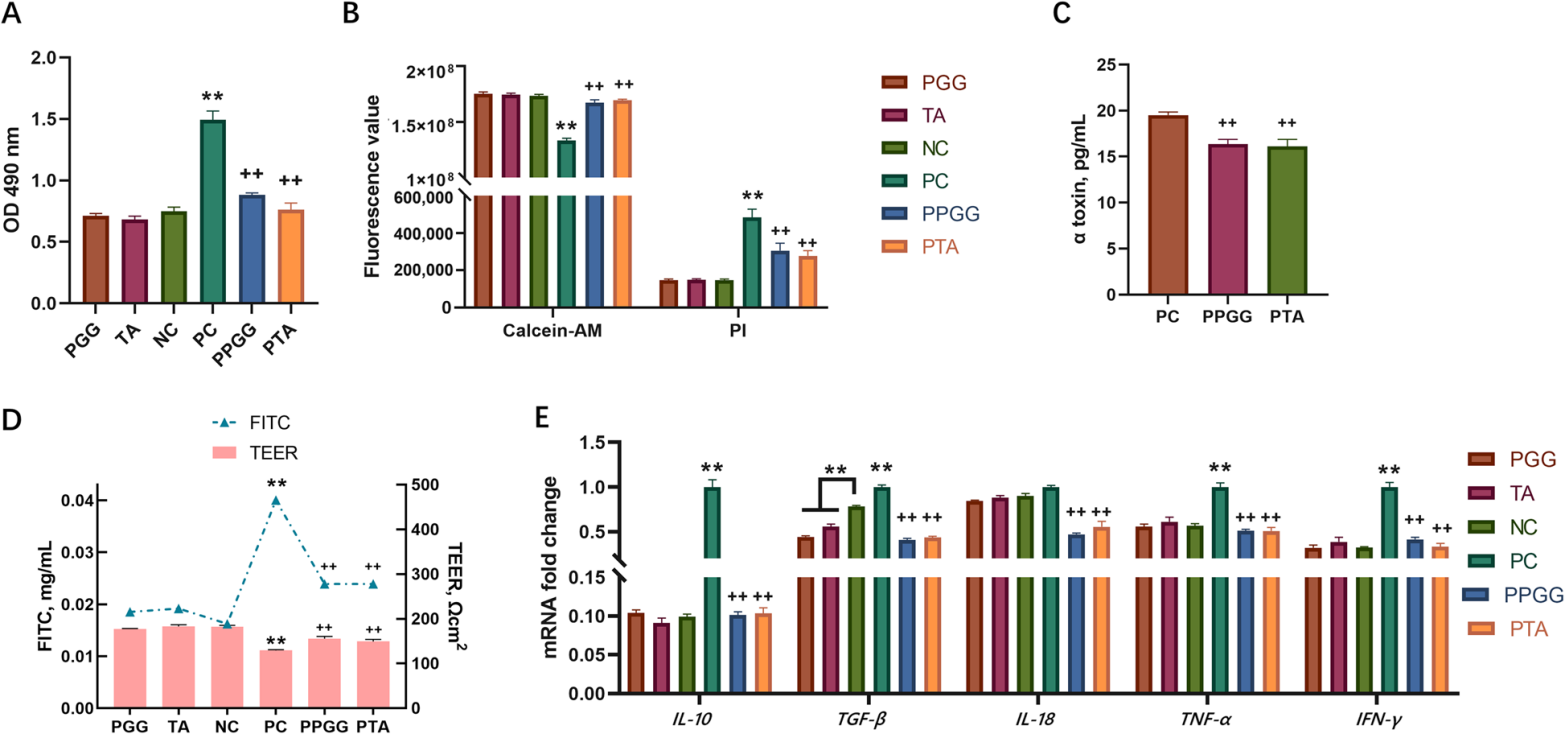
Protective effects of PGG and TA on intestinal epithelial cells infected with *Clostridium perfringens.* **A** Effects of PGG, TA, and *C. perfringens* treatment on lactate dehydrogenase activity in intestinal epithelial cells, *n* = 8. **B** Effects of PGG, TA, and *C. perfringens* treatment on intestinal epithelial cell viability, *n* = 12. **C** Effects of PGG and TA treatment on α toxin content in the intestinal epithelial cell serum after *C. perfringens* treatment, *n* = 6. **D** Effects of PGG, TA, and *C. perfringens* treatment on the barrier function of intestinal epithelial cells, *n* = 4. **E** Effects of PGG, TA, and *C. perfringens* treatment on the mRNA expression of inflammatory factors in intestinal epithelial cells, *n* = 6. PGG: PGG treatment + intestinal epithelial cells; TA: TA treatment + intestinal epithelial cells; NC: negative control group with intestinal epithelial cells only; PC: *C. perfringens* + intestinal epithelial cells; PPGG: *C. perfringens* + intestinal epithelial cells + PGG; PTA: *C. perfringens* + intestinal epithelial cells + TA. ^**^P < 0.01 compared with the NC group; ^++^*P* < 0.01 compared with the PC group; PGG, pentagalloylglucose; TA, tannic acid

PGG and TA did not significantly affect the lactate dehydrogenase (LDH) activity in the cell supernatant, compared with the NC group (Fig. 8A). However, the LDH level in the PC group was notably higher than in the NC group (*P* < 0.01). Notably, LDH activity was reduced in the PPGG and PTA groups than in the PC group (*P* < 0.01). Additionally, a significant decrease in the fluorescence intensity of calcein-AM—indicating cell activity—was observed in the PC group (*P* < 0.01; Fig. 8B). In contrast, the fluorescence intensity of PI—indicating cell death—significantly increased compared with that in the NC group (*P* < 0.01). Meanwhile, the PPGG and PTA groups exhibited significantly higher calcein-AM fluorescence values and significantly lower PI fluorescence values than the PC group (*P* < 0.01).

Within the supernatant of cells in the PGG and TA groups, α toxin levels were significantly decreased compared with those in the PC group (*P* < 0.01; Fig. 8C). The TEER results for the intestinal epithelium indicated that compared with the NC group, PGG and TA did not significantly affect the barrier function of the intestinal epithelium; however, the TEER value in the PC group significantly decreased (*P* < 0.01; Fig. 8D). In contrast, the TEER values in the PPGG and PTA groups were significantly higher than those in the PC group (*P* < 0.01). Similarly, the FITC level in the lower compartment of the Transwell was significantly higher in the PC group than in the NC group (*P* < 0.01). The FITC level in the lower compartment was significantly lower in the PPGG and PTA groups than in the PC group (*P* < 0.01).

The level of transforming growth factor (TGF)-β in the PGG and TA groups was significantly lower than in the NC group (*P* < 0.01; Fig. 8E). Moreover, the mRNA expression of interleukin 10 (*IL-10*), *TGF*-*β,* interferon-γ (*IFN*-γ), and tissue necrosis factor-α (*TNF*-α) was upregulated in the PC group than in the NC group (*P* < 0.01). Conversely, the expression of *IL-10*, *IL-18*, *TNF*-α, *TGF*-*β*, and *IFN*-γ was significantly downregulated in the PPGG and PTA groups than in the PC group (*P* < 0.01).

### Protective effects of PGG and TA on *C. perfringens*-infected mice

**Fig. 9:**
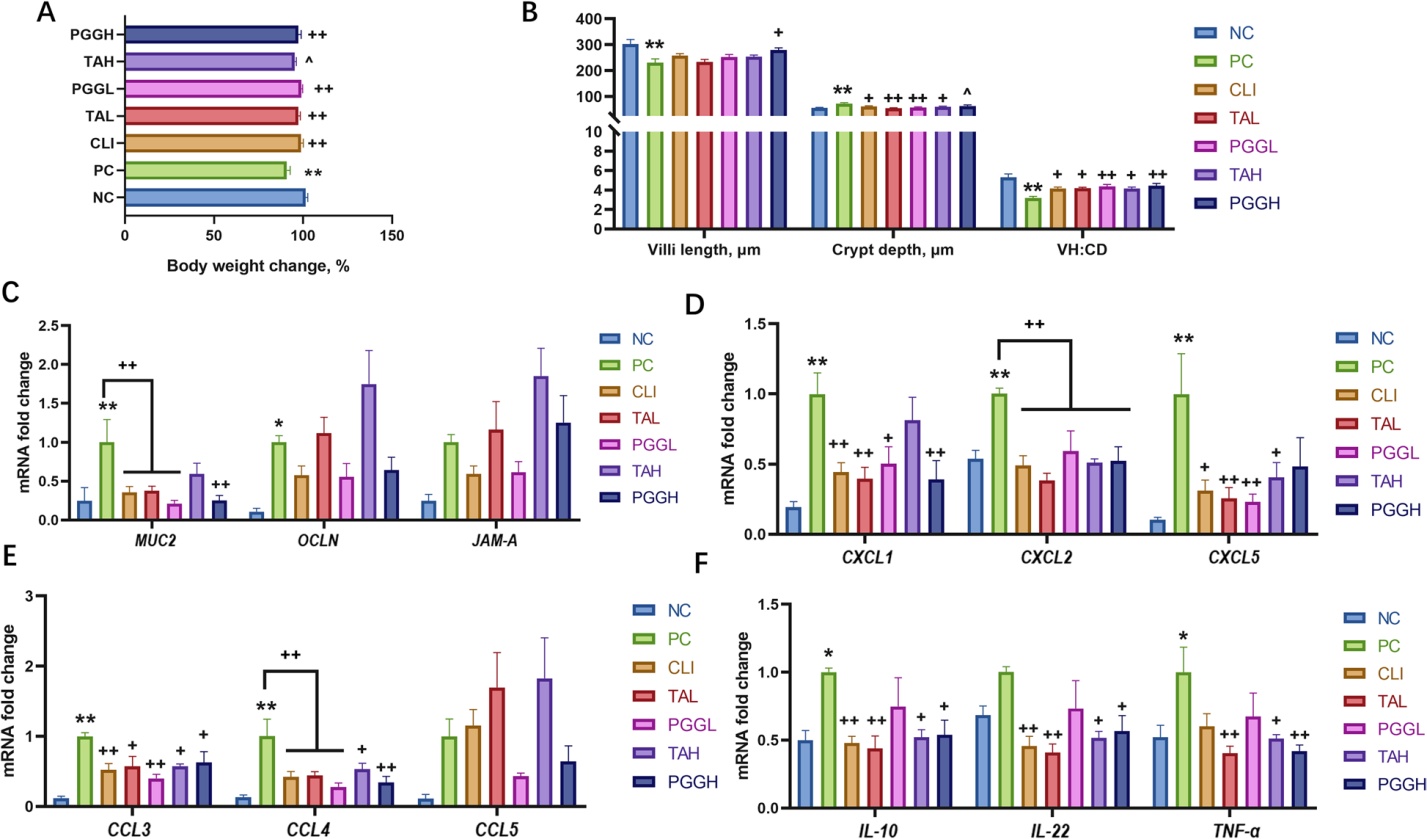
The alleviating effects of PGG and TA on *C. perfringens* infected mice. **A** Changes in body weight after the attack. **B** Statistical analysis of ileal villi morphology. **C–F** Expression of genes related to the ileal intestinal barrier and inflammation. ^*^P < 0.05, ^**^P < 0.01 compared with the NC group; ^^^0.05 ≤ *P* < 0.1, ^+^*P* < 0.05, ^++^*P* < 0.01 compared with the PC group. PGG, pentagalloylglucose; TA, tannic acid. *n* = 6

In vivo experiments validated the protective effects of PGG and TA against *C. perfringens* infection in mice. Body weight changes in *C. perfringens*-infected mice were significantly reduced compared with those in the NC group (*P* < 0.01; Fig. 9A). Compared with the PC group, the CLI, TAL, PGGL, and PGGH groups (all *P* < 0.01) exhibited significant increases in body weight changes, and the TAH group has a tendency to increase body weight changes (*P* < 0.1).

Morphometric analysis of the ileal histology revealed that compared with the NC group, the PC group had significantly reduced villi height (*P* < 0.01), decreased villus height-to-crypt depth ratio (VH:CD, *P* < 0.01), and increased crypt depth (*P* < 0.01; Fig. 9B). Meanwhile, compared with the PC group, the PGGH group exhibited significantly improved villi height (*P* < 0.05), while the CLI, TAH (both *P* < 0.05), TAL, and PGGL (both *P* < 0.01) groups exhibited significant reductions in crypt depth. Additionally, administering antibiotics (*P* < 0.05), PGG (*P* < 0.01), or TA (*P* < 0.05) significantly restored the ileal VH:CD ratio.

Quantitative analysis of ileal tight junction-related genes (Fig. 9C) revealed that the PC group had significantly upregulated mucin 2 (*MUC2*, *P* < 0.01) and occludin (*OCLN*, *P* < 0.05) expression compared with the NC group. CLI, TAL, PGGL, and PGGH groups significantly reduced *MUC2* expression (*P* < 0.01).

Assessment of ileal inflammatory gene expression (Fig. 9D–F) demonstrated that the PC group had significantly upregulated expression of chemokine (C-X-C motif) ligand 1 (*CXCL1*), *CXCL2*, *CXCL5*, chemokine (C-C motif) ligand 3 (*CCL3*), *CCL4* (all *P* < 0.01), *IL-10*, and TNF-α (all *P* < 0.05), compared with the NC group. Compared with the PC group, the CLI group exhibited significant downregulation of *CXCL1*, *CXCL2*, *CCL3*, *CCL4*, *IL-10*, *IL-22* (*P* < 0.01) and *CXCL5* (*P* < 0.05). The TAL group downregulated the expression of *CXCL1*, *CXCL2*, *CXCL5*, *CCL4*, *IL-10*, *IL-22*, *TNF-α* (all *P* < 0.01), and *CCL3* (*P* < 0.05). The PGGL group exhibited downregulated expression of *CXCL2*, *CXCL5*, *CCL3*, *CCL4* (all *P* < 0.01), and *CXCL1* (*P* < 0.05); while the TAH group downregulated the expression of *CXCL2* (*P* < 0.01), *CXCL5*, *CCL3*, *CCL4, IL-10*, *IL-22* and *TNF-α* (all *P* < 0.05). Furthermore, the PGGH group downregulated  the expression of *CXCL1*, *CXCL2*, *CCL4*, *TNF-α* (all *P* < 0.01), *CCL3, IL-10*, and *IL-22* (both *P* < 0.01).

## Discussion

### PGG and TA inhibit *C. perfringens* proliferation by disrupting the bacterial cell membrane

Measuring the concentration of K^+^ and Mg^2+^ in culture supernatant provides indirect information regarding the bacterial cell membrane integrity and the survival status [19, 20]. PGG and TA exert toxic effects on *C. perfringens*, causing release of intracellular K^+^ and Mg^2+^ into the supernatant. This suggests that PGG and TA increase the bacteria’s membrane permeability, causing a significant efflux of intracellular inorganic salt ions. This is consistent with previous reports suggesting that the antimicrobial activity of tannins is closely associated with their interaction with bacterial membranes [21, 22].

The combined transcriptomic and metabolomic analysis in the current study revealed that PGG and TA downregulated the peptidoglycan biosynthetic pathway. This may disrupt the integrity of Gram-positive bacteria, which possess a distinct cell wall structure comprising a single lipid membrane surrounded by a thick outer layer of peptidoglycan, which serves as a protective barrier [23]. PGG and TA also interfered with pyrimidine metabolism. Pyrimidine nucleotides not only serve as precursors for RNA and DNA but are also vital in the biosynthesis of cell membrane components, including peptidoglycans and extracellular polysaccharides [24]. Hence, PGG and TA may affect the synthesis of certain cell membrane components. The integrated transcriptomic and metabolomic analyses suggest that tannins disrupt bacterial cell membranes by altering pyrimidine metabolism and downregulating peptidoglycan synthesis.

### PGG and TA inhibit *C. perfringens* proliferation through amino acid restriction

Transcriptomic analysis further indicated that PGG and TA also downregulate genes associated with amino acid synthesis; specifically, lysine, arginine, phenylalanine, ornithine, and tyrosine levels were markedly lower than those in the control group. This suggests an imbalance in the intracellular amino acid status, impacting myriad cellular pathways, including ribosomal biosynthesis. In bacteria, ribosome biosynthesis is highly cost-intensive and a major growth-limiting factor, necessitating its synchronization to increase bacterial fitness [25]. Thus, during nutritional deprivation or under external stress, bacteria adjust the number of active ribosomes, block the overexpression of high-abundance ribosomal proteins, and redirect limited resources to synthesize other stress-relieving proteins to avoid wasting resources [26]. Indeed, PGG and TA downregulated genes associated with ribosome biosynthesis, specifically with the biosynthesis of tRNA bound to amino acids.

Taken together, PGG and TA treatment directly interfered with the biosynthesis and metabolism of amino acids in *C. perfringens*, resulting in deficiencies and imbalances within the bacterium. In response, the bacterium uses signaling to balance its ribosome-to-substrate ratio and achieve the maximum degree of saturation [26, 27]. That is, bacteria adjust their biosynthesis of ribosomes and tRNAs to align with protein synthesis needs and resist external stress [28–30]. Hence, tannins exert antibacterial effects through a coordinated mechanism involving amino acid restriction and disruption of ribosomal homeostasis, ultimately compromising bacterial adaptive capacity.

### PGG and TA inhibit *C. perfringens* proliferation through impaired energy metabolism

Pyruvate is a crucial intermediate in metabolic processes [31]. Pyruvate metabolism is evolutionarily conserved and vital in carbon homeostasis, facilitating glycolysis and oxidative phosphorylation [32]. In the current study, PGG and TA were found to interfere with pyruvate metabolism in *C. perfringens*. Further analysis of the related products during pyruvate metabolism revealed that the levels of citric, succinic, and malic acid were markedly reduced by PGG and TA treatment. This suggests that PGG and TA disrupt the bacterium's energy metabolism by affecting pyruvate metabolism. Similarly, the broad-spectrum anti-infective drug thiazolide nitazoxanide adversely affects anaerobic bacteria proliferation by inhibiting pyruvate and thus impairing energy metabolism [33, 34].

Purines are essential for cellular processes, including energy metabolism, genetic material encoding, and cell signaling across all organisms [35, 36]. PGG and TA affect purine and pyrimidine metabolism in *C. perfringens*, potentially impacting energy metabolism. Collectively, these findings suggest that by suppressing pyruvate metabolism, PGG and TA induce a cascade collapse in downstream intermediates (citrate, succinate, malate), directly crippling oxidative phosphorylation and adenosine triphosphate (ATP) synthesis. This primary energy crisis is compounded by the interference with purine/pyrimidine metabolism.

### PGG and TA affect *C. perfringens* proliferation by interfering with its sensing and transport mechanisms in response to external stimuli

Bacteria are constantly exposed to stimuli from the external environment and must respond appropriately. Bacteria use the two-component system as a signaling mechanism to control pathogenesis, stress responses, and symbiotic interactions in response to environmental factors [37, 38]. Some bacteria (e.g., staphylococci, streptococci, and *Clostridium *spp.) have evolved membrane protein complexes to sense changes in the external environment. These complexes comprise the ATP-binding cassette (ABC) transporter system and a two-component system [39, 40]. As the largest and oldest protein superfamily, ABC transporters shuttle uses the binding and hydrolysis of ATP to transport myriad substrates across cell membranes, including vitamins, steroids, ions, peptides, proteins, lipids, polysaccharides, and exogenous substances [41]. In the current study, PGG and TA were found to interfere with the metabolism of glycerol esters, glyoxylate, dicarboxylate, nicotinic acid, nicotinamide, thiamine, butyrate, and vitamin B_6_ in *C. perfringens*. This may be related to their influence on ABC transporters. Specifically, PGG and TA may hinder the bacteria's ability to respond to environmental cues by interfering with these signaling systems, placing the bacteria at a survival disadvantage.

Bacteria communicate using quorum sensing, involving the release, response to, and detection of diffusible signaling molecules [42]. Quorum sensing is important for biofilm formation, motility, extracellular polysaccharide production, and chemotaxis [43, 44]. Thus, PGG and TA may impair quorum sensing and disrupt the bacteria’s perception of external environmental stimuli, impacting *C. perfringens* proliferation.

In summary, tannins may disrupt the two-component signaling system and quorum sensing, blocking bacterial recognition of stress signals and coordinating population behaviors, which weakens their adaptive survival strategies and impairs environmental perception. This further suppresses ABC transporter functionality, hindering the uptake of critical metabolites and energy substrates, ultimately leading to coenzyme deficiency, collapse of metabolic pathways, and exacerbated cell death.

### PGG and TA protect the intestines by inhibiting α toxin production and binding

The toxins released by *C. perfringens* induce a wide range of tissue toxicity. *C. perfringens* encodes at least two distinct quorum-sensing systems: Agr-like and LuxS. These systems are important regulators of virulence and toxin production [45, 46]. The two-component VirR/VirS system positively regulates several *C. perfringens* toxins, including α, β2, and NetB toxins [47, 48]. In this study, PGG and TA were found to bind to α toxin and inhibit its production. They also downregulated toxin-encoding genes. Therefore, PGG and TA may inhibit the synthesis of related toxins of *C. perfringens* by interfering with the two-component system and quorum sensing.

The α toxin protein can bind polyphenols through three primary groups of intermolecular interactions [49]: covalent or non-covalent, soluble or insoluble, and specific or non-specific [50]. In proteins’ secondary structures, non-covalent interactions can lead to partial conformational changes [51]. Hence, PGG and TA binding α toxin may alter the spatial structure of the α-toxin and cause it to precipitate. However, compared with PGG, TA exhibited a lower binding energy with α toxin, indicating that it binds more strongly to the α toxin. The strength of tannin–protein binding is related to tannin’s molecular weight [52, 53]; that is, the larger number of binding sites on TA than on PGG improves binding. This also suggests the potential of structurally complex tannins as effective inhibitors of bacterial toxins.

The intestines form a crucial barrier, preventing pathogens and toxins from crossing the mucosa and entering the body while activating the immune system to combat these threats. As PGG and TA inhibit *C. perfringens* and α toxin, their protective effects on the intestines were further investigated. Co-culturing *C. perfringens* with intestinal epithelial cells resulted in a significant inflammatory response and cell death, consistent with previous findings [54, 55]. Meanwhile, treatment with PGG or TA reduced the expression of inflammatory cytokines, mitigating the excessive immune response triggered by *C. perfringens*. This anti-inflammatory response was confirmed in a mouse model. Similarly, the anti-inflammatory effects of tannins have been confirmed in other inflammation models. [56, 57].

Tight junction complexes connect intestinal epithelial cells, regulating the intestinal barrier's permeability. Pathogens, including *C. perfringens*, can disrupt this barrier [58], inducing the inflammatory response and further compromising barrier function through host cell signaling cascades [59, 60]. PGG and TA protect intestinal epithelial barrier function by inhibiting *C. perfringens* and α toxin production, and modulating inflammatory responses.

This study demonstrated that PGG and TA integrate antibacterial, antitoxin, and host-directed anti-inflammatory actions to against *C. perfringens*-induced intestinal inflammation. This provides a novel strategy to combat enteric infections without exacerbating dysbiosis or antibiotic resistance.

## Conclusions

PGG and TA inhibit *C. perfringens* proliferation and pathogenicity through a multi-target mechanism: (1) disrupt bacterial membrane integrity; (2) interfere with amino acid metabolism and ribosomal homeostasis to restrict bacterial resource allocation; (3) suppress pyruvate metabolism, leading to energy metabolism collapse; (4) block the two-component signaling system and quorum sensing to weaken environmental adaptability; and (5) reduce α toxin synthesis, and neutralize α toxin. Additionally, PGG and TA protect intestinal barrier function and modulate inflammatory responses. This antibacterial–antitoxin–anti-inflammatory triad mechanism of tannins highlights a novel strategy for antibiotic-free control of intestinal infections.

In summary, tannins have unique benefits for controlling foodborne pathogens, sustainable livestock farming, and intestinal health. Future research should focus on formulation innovations and combinatorial strategies to enhance their application efficiency and develop green bioprotection technologies.

## Supplementary Information

Supplementary Material 1. Supplementary Fig. 1 Schematic representation of the PGG and TA structures. PGG, pentagalloylglucose; TA, tannic acid.

Supplementary Material 2. Supplementary Fig. 2 Effects of PGG and TA on *cpa* and *cpb2* genes of *Clostridium perfringens*.

Supplementary Material 3. Supplementary Fig. 3 Analysis of PGG versus TA differential genes and differential metabolites.

## Data Availability

The raw reads of all samples were deposited in the Sequence Read Archive (SRA) of the National Center for Biotechnology Information (NCBI) as FASTQ files with SRP accession number PRJNA1184444. The raw metabolomics data for *C. perfringens* can be accessed on the MetaboLights database under the accession number MTBLS11631. All data produced and analyzed in this research can be obtained from the corresponding author upon request.

## Abbreviations

ATP: Adenosine triphosphate
CCL3: Chemokine (C-C motif) ligand 3
CCL4: Chemokine (C-C motif) ligand 4
CCL5: Chemokine (C-C motif) ligand 5
CXCL1: Chemokine (C-X-C motif) ligand 1
CXCL2: Chemokine (C-X-C motif) ligand 2
CXCL5: Chemokine (C-X-C motif) ligand 5
ELISA: Enzyme-linked immunosorbent assay
GAPDH: Glyceraldehyde-3-phosphate dehydrogenase
JAM-A: Junctional adhesion molecule A
IL-10: Interleukin-10
IL-22: Interleukin-22
KEGG: Kyoto Encyclopedia of Genes and Genome
MUC2: Mucin 2
NE: Necrotic enteritis
OCLD: Occluding
PBS: Phosphate buffer saline
PGG: Pentagalloylglucose
PI: Propidium iodide
TA: Tannic acid
TEER: Trans-epithelial electrical resistance
TNF-α: Tumor necrosis factor-alpha
