# Supplementary material for: Integrated transcriptomics and metabolomics reveal multi-target mechanisms of tannins against Clostridium perfringens and necrotic enteritis
Source: J Anim Sci Biotechnol. 2025 Jul 14;16:98. doi: 10.1186/s40104-025-01228-3 (PMC12257667; doi:10.1186/s40104-025-01228-3)
Supplement: Supplementary file 1 — Supplementary Material 1. Supplementary Fig. 1 Schematic representation of the PGG and TA structures. PGG, pentagalloylglucose; TA, tannic acid. [file 40104_2025_1228_MOESM1_ESM.docx]

**Supplementary Figure 1**

**
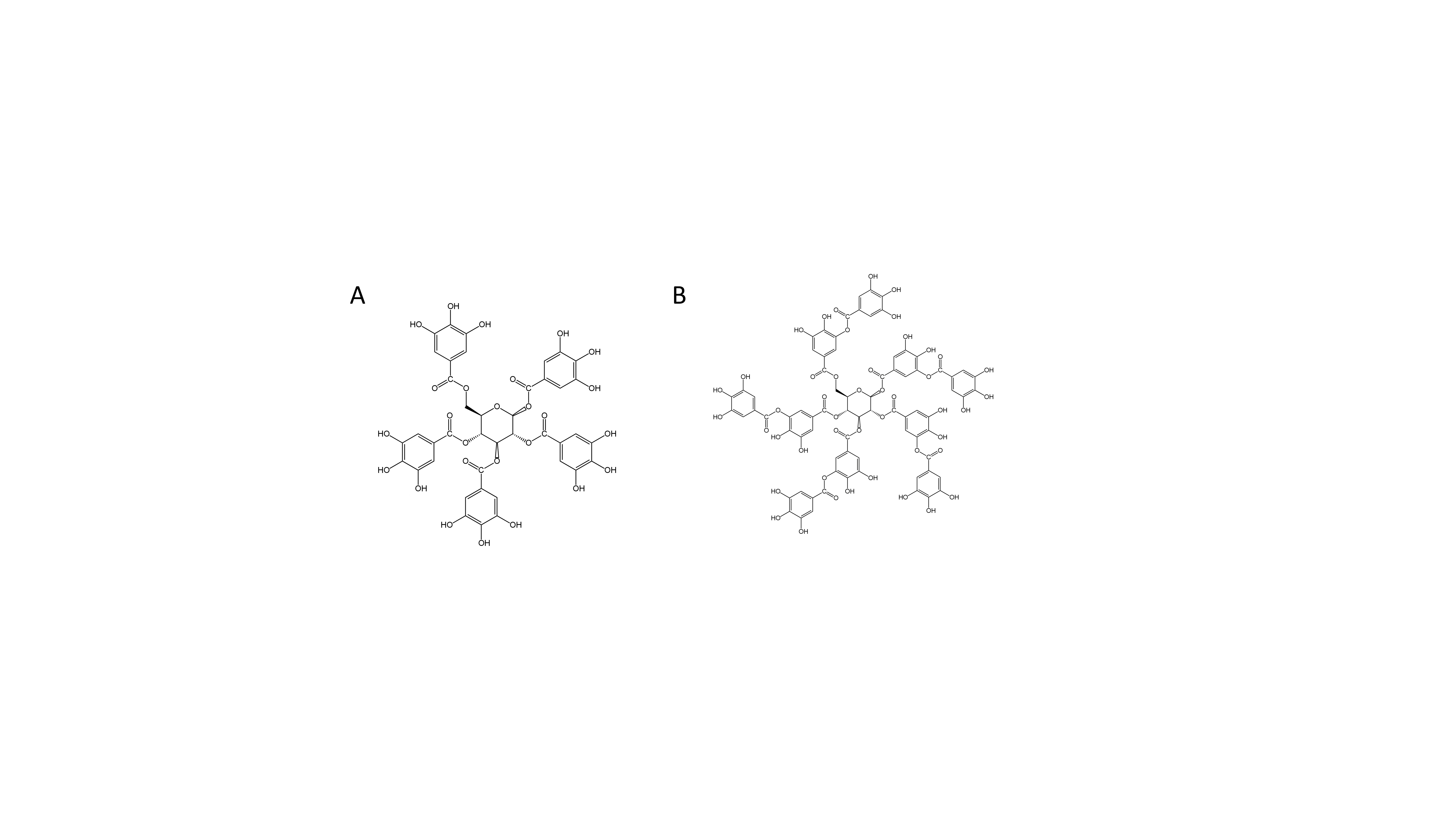
**

**Supplementary Fig.** **1** Schematic representation of the PGG and TA structures. PGG, pentagalloylglucose; TA, tannic acid.
