# Supplementary material for: Integrated transcriptomics and metabolomics reveal multi-target mechanisms of tannins against Clostridium perfringens and necrotic enteritis
Source: J Anim Sci Biotechnol. 2025 Jul 14;16:98. doi: 10.1186/s40104-025-01228-3 (PMC12257667; doi:10.1186/s40104-025-01228-3)
Supplement: Supplementary file 2 — Supplementary Material 2. Supplementary Fig. 2 Effects of PGG and TA on cpa and cpb2 genes of Clostridium perfringens. [file 40104_2025_1228_MOESM2_ESM.docx]

**Supplementary Figure 2**


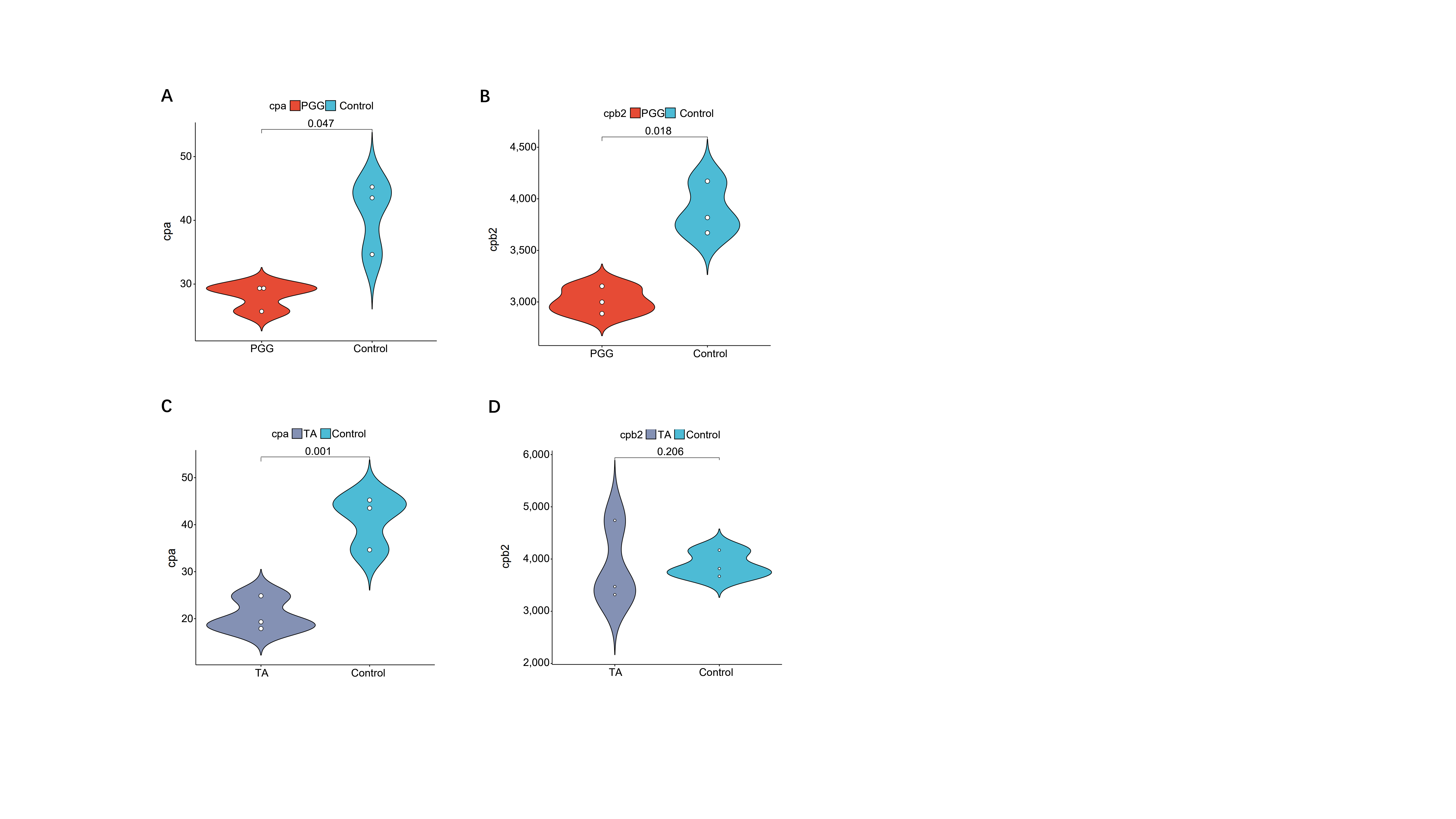


**Supplementary Figure 2.** Effects of PGG and TA on *cpa* and *cpb2* genes of *Clostridium perfringens*. A, B. Effects of PGG on *cpa* and *cpb2* gene expression, respectively. C, D. Effects of TA on *cpa* and *cpb2* gene expression, respectively. PGG, pentagalloylglucose; TA, tannic acid.
