# Supplementary material for: Integrated transcriptomics and metabolomics reveal multi-target mechanisms of tannins against Clostridium perfringens and necrotic enteritis
Source: J Anim Sci Biotechnol. 2025 Jul 14;16:98. doi: 10.1186/s40104-025-01228-3 (PMC12257667; doi:10.1186/s40104-025-01228-3)
Supplement: Supplementary file 3 — Supplementary Material 3. Supplementary Fig. 3 Analysis of PGG versus TA differential genes and differential metabolites. [file 40104_2025_1228_MOESM3_ESM.docx]

Compared to the TA-treated group, 15 genes were down-regulated and 17 genes were up-regulated in the PGG-treated group. The up-regulated genes include the arginine-ornithine reverse transporter protein arcD, ribosome-related genes rimP and ylqF, and the flagellin-related gene filB. The down-regulated genes mainly include rpsT and pstC. This suggests that TA inhibits the expression of genes related to protein synthesis, transport, and flagellin more than PGG does. Additionally, Kyoto Encyclopedia of Genes and Genomes (KEGG) enrichment analysis of the differential genes revealed that, compared to TA, PGG up-regulates genes involved in purine metabolism, thiamine metabolism, and oxidative phosphorylation. Conversely, it down-regulates genes associated with ABC transporters, ribosomes, quorum sensing, the two-component system, and DNA replication.

Next, the metabolomics of PGG and TA were analyzed, revealing 255 differential metabolites in PGG compared to TA. The down-regulated metabolites in the PGG group included xanthylic acid, ellagic acid, ethyl gallate, methyl gallate, hypoxanthine, and cupressufavone, which inhibit inflammation, as well as Patulin. The up-regulated metabolites were focused on glucuronolactone, ADP, and others. When the differential metabolites were categorized, they were mainly concentrated in carboxylic acids and derivatives, benzene and substituted derivatives, and glycerophospholipids. KEGG enrichment analysis revealed that the pathways of differential metabolites were concentrated in nucleotide metabolism (pyrimidine and purine metabolism), lipid metabolism (including glycerophospholipid and sphingolipid metabolism), cofactor biosynthesis, amino acid metabolism (lysine degradation, valine, leucine, and isoleucine degradation, arginine and proline metabolism), aminoacyl-tRNA biosynthesis, and energy metabolism (oxidative phosphorylation and quorum sensing).


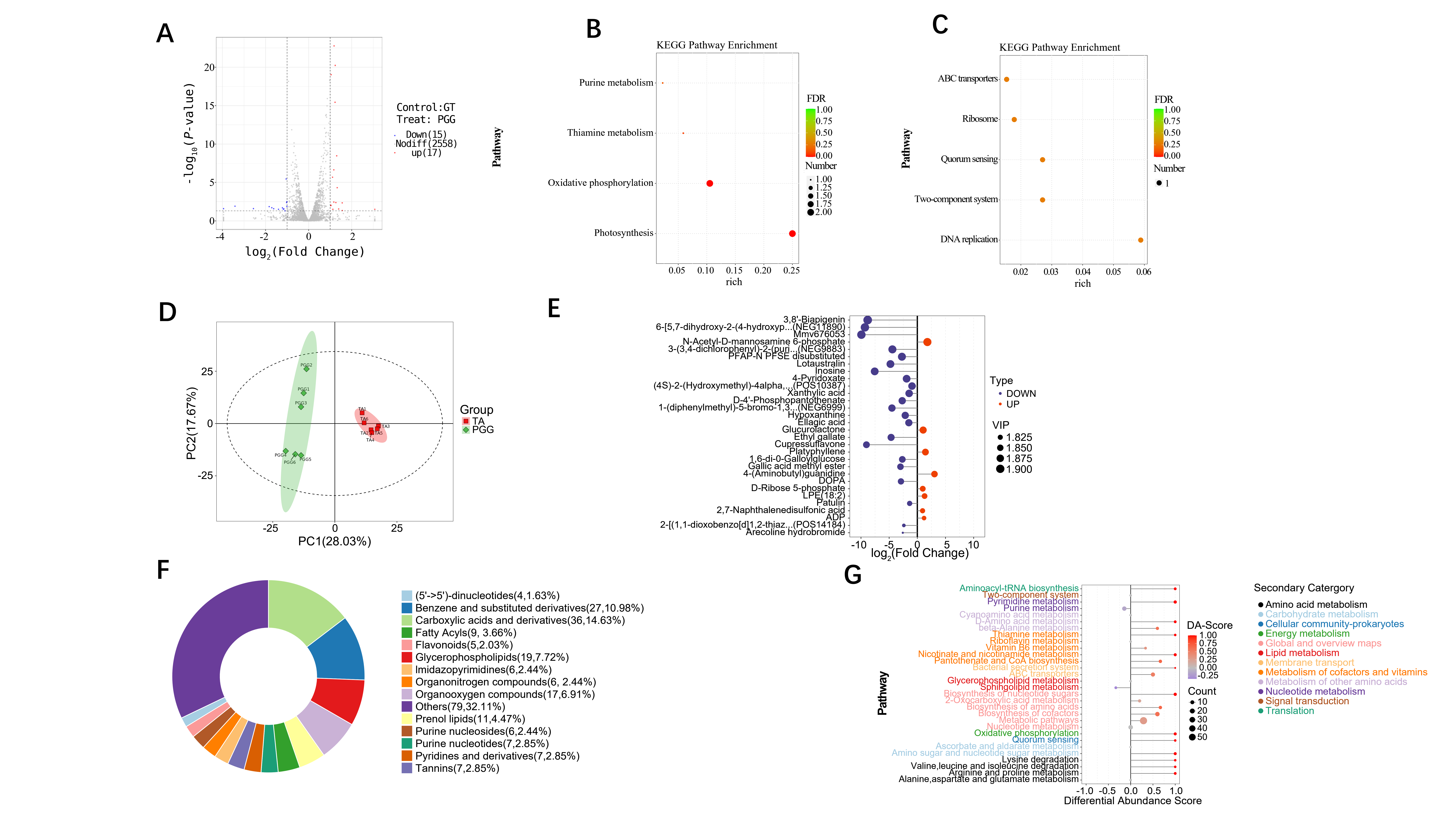
**Supplementary Figure 3.** Analysis of PGG versus TA differential genes and differential metabolites. **A**. The figure of differential gene volcanoes. **B**. The figure of up-regulated pathways enriched for differential genes. **C**. The figure of down-regulated pathways enriched for differential genes. **D**. The figure of PCA of differential metabolites. **E**. The figure of differential metabolites of higher importance. **F**. The figure of categorization of differential metabolites. **G**. The figure of pathways enriched for differential metabolites. PGG, pentagalloylglucose; TA, tannic acid.
